# One-Step Synthesis of Polyethyleneimine-Grafted Styrene-Maleic Anhydride Copolymer Adsorbents for Effective Adsorption of Anionic Dyes

**DOI:** 10.3390/molecules29081887

**Published:** 2024-04-21

**Authors:** Yao Xu, Qinwen Wang, Yuanbo Wang, Falu Hu, Bin Sun, Tingting Gao, Guowei Zhou

**Affiliations:** 1 Key Laboratory of Fine Chemicals in Universities of Shandong, Jinan Engineering Laboratory for Multi-Scale Functional Materials, School of Chemistry and Chemical Engineering, Qilu University of Technology (Shandong Academy of Sciences), Jinan 250353, China; 10431210232@stu.qlu.edu.cn (Y.X.); 10431220334@stu.qlu.edu.cn (Q.W.); binsun@qlu.edu.cn (B.S.); gwzhou@qlu.edu.cn (G.Z.); 2 Shandong Land and Space Ecological Restoration Center, Jinan 250014, China; wybdhzz@shandong.cn; 3 Shandong Laboratory of Advanced Materials and Green Manufacturing at Yantai, Yantai 264006, China

**Keywords:** adsorption, dyes, graft-modification, styrene-maleic anhydride copolymer (SMA), polyethyleneimine (PEI)

## Abstract

Wastewater containing organic dyes has become one of the important challenges in water treatment due to its high salt content and resistance to natural degradation. In this work, a novelty adsorbent, PEI-SMA, was prepared by grafting polyethyleneimine (PEI) onto styrene-maleic anhydride copolymer (SMA) through an amidation reaction. The various factors, such as pH, adsorbent dosage, contact time, dye concentration, and temperature, which may affect the adsorption of PEI-SMA for Reactive Black 5 (RB5), were systematically investigated by static adsorption experiments. The adsorption process of PEI-SMA for RB5 was more consistent with the Langmuir isotherm model and the pseudo-second-order model, suggesting a single-layer chemisorption. PEI-SMA exhibits excellent adsorption performance for RB5 dye, with a maximum adsorption capacity of 1749.19 mg g^–1^ at pH = 2. Additionally, PEI-SMA exhibited highly efficient RB5 competitive adsorption against coexisting Cl^−^ and SO_4_^2−^ ions and cationic dyes. The adsorption mechanism was explored, and it can be explained as the synergistic effect of electrostatic interaction, hydrogen bonding and π–π interaction. This study demonstrates that PEI-SMA could act as a high performance and promising candidate for the effective adsorption of anionic dyes from aqueous solutions.

## 1. Introduction

Organic dyes are widely applied in textile printing and dyeing, coatings, plastics, leather, optoelectronic communication, food and other fields and are undoubtedly one of the indispensable chemicals in our daily lives [1,2,3]. However, water pollution caused by the uncontrolled discharge of organic dyes during the production represents a threat to the ecological environment and biological survival [4]. The complex aromatic structure of dyes makes them highly stable, heat and light resistant, as well as poorly biodegradable [5]. The direct discharge of dyes into the water environment can prevent the penetration of sunlight and reduce the photosynthesis of aquatic plants [6]. In addition, dyes are inherently toxic and carcinogenic, with the potential to accumulate in animals and cause serious health problems [7,8]. Therefore, it is of great importance and urgency to achieve efficient treatment of dye wastewater.

Various technologies have been applied for dye wastewater treatment, including adsorption, ion exchange, membrane filtration, photocatalytic degradation, biodegradation, coagulation/flocculation, precipitation, permeation, etc. [9,10,11,12,13]. Among these techniques, adsorption is considered the easiest and most promising process for removing dyes from wastewater due to its ease of operation, good economy, high removal efficiency, reusability, and lack of toxic intermediates [14].

Currently, activated carbon and adsorption resin are the most commonly used dye adsorbents in the industry, but there are still problems, such as low adsorption capacity and poor adsorption selectivity [4,14]. In order to develop more efficient and low-cost dye adsorbents, other carbon-based materials [15,16], metal oxides [17], polymeric materials [18,19,20], metal organic frameworks (MOFs) [21,22], biosorbents [23,24], and organic/inorganic composite materials have been widely studied [25]. The cationic polyethyleneimine (PEI) polymer possesses a large amount of reactive amine groups (–NH–, –NH_2_), which are prone to protonation at pH < 10, making the PEI positively charged and able to adsorb anions through strong electrostatic interactions [26]. In addition, PEI has the advantages of low cost, non-toxicity, and good biocompatibility in water [27]. However, owing to the water-soluble nature of PEI, its direct use as an adsorbent will make it difficult to separate and recover from the adsorbent solution. Therefore, PEI usually requires chemical modification, such as cross-linking with other chemicals or being immobilized on other insoluble solid substrates. Hu et al. synthesized a new adsorbent by grafting PEI onto a UiO-66-NH_2_ MOFs with glutaraldehyde as a crosslinking agent, and the theoretical maximum adsorption capacities toward Methyl Orange (MO) and Pb(II) reach 497.51 and 692.80 mg g^–1^, respectively [28]. Wang et al. prepared a PEI-functionalized chitosan (CS) aerogel with epichlorohydrin as a crosslinking agent, and the theoretical maximum adsorption capacity toward Cr(Ⅵ) was 445.29 mg g^–1^ [29]. Usman et al. presented the synthesis of PEI-based sponges through the ice-templating method via 1,4-butanediol diglycidyl ether (BDDE) as the crosslinker, and the adsorbent showed a higher adsorption capacity towards Congo Red (CR) [30]. Obviously, PEI grafting or crosslinking is an effective way to overcome its water solubility problem and fully utilize the active amine groups on PEI to adsorb dyes. However, the above carriers or backbone polymers for grafting PEI usually need an extra crosslinker and occupy the active amine sites, which can decrease the adsorption capacity for dyes. Therefore, in order to achieve a greater adsorption capacity, it is necessary to find a support that can graft PEI directly.

SMA contains hydrophobic phenyl and reactive anhydride groups. The anhydride groups can easily react with chemicals such as water, alcohols, and amines containing –OH/–NH_2_ groups, providing a variety of options for the chemical modification and functionalization of SMA copolymers [31,32]. These unique properties make SMA a powerful candidate for a hydrophobic carrier for immobilizing or crosslinking amine compounds, such as PEI, to prepare amphiphilic amine-rich polymers for dye adsorption.

This work used commercially available alternating SMA and PEI as raw materials to prepare a PEI graft-modified SMA (PEI-SMA) polymer adsorbent through a one-step amidation reaction without an additional crosslinking agent. The aim was to optimize the structural monomer ratio of PEI/SMA in PEI-SMA to introduce more reactive amine groups and further improve its adsorption performance for anionic dyes. The adsorption behavior of PEI-SMA on anionic dyes represented by RB5 was systematically investigated, and the effects of coexisting ions and simulated mixed dye solutions on the removal of RB5 by PEI-SMA were examined. Furthermore, the regeneration adsorption performance of PEI-SMA was also tested.

## 2. Results and Discussion

### 2.1. PEI-SMA Characterization

**Figure 1:**
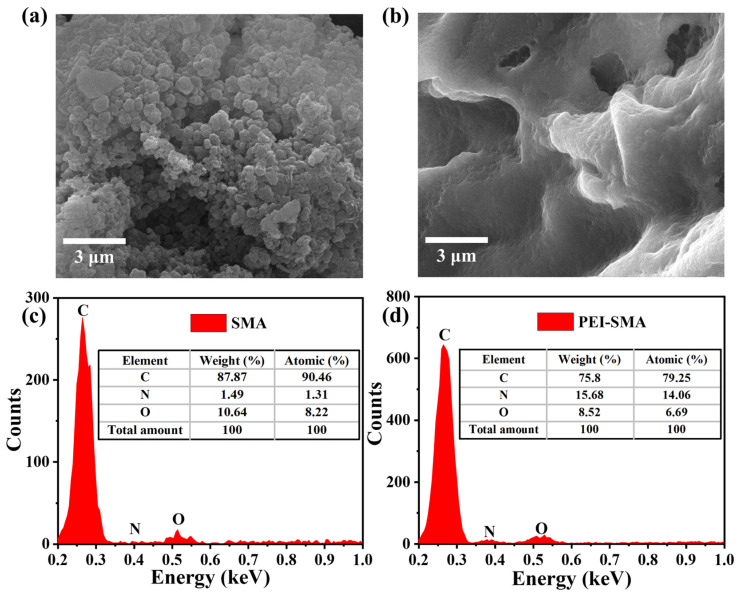
SEM images of (**a**) SMA and (**b**) PEI-SMA, EDS spectra of (**c**) SMA and (**d**) PEI-SMA.

The surface morphologies of SMA and PEI-SMA are exhibited in Figure 1a,b. The SEM shows that the SMA consists of a pile-up of small spheres with rough surfaces, while the surface of PEI-SMA exhibits a smoother structure than SMA, and meanwhile, the small spherical stacking structure is transformed into a laminar stacking structure. As illustrated in Figure 1c,d, the EDS data demonstrate that the surface N content of PEI-SMA (15.68%) is much higher than that of SMA (1.49%), which may come from the amidation reaction of SMA and PEI. The above changes in microscopic morphology and N content confirm the successful grafting of PEI onto the surface SMA.

In order to investigate the effect of modification on the specific surface area and porosity of PEI-SMA, the adsorption isotherms of SMA and PEI-SMA were determined using the N_2_ adsorption–desorption method (Figure S1). The adsorption–desorption isotherms of the SMA and PEI-SMA are of type Ⅱ, characteristic of typical non-porous or microporous materials. The BET surface areas of the SMA and PEI-SMA are 16.4188 m^2^/g and 0.2266 m^2^/g, the total pore volumes are 0.014067 cm^3^/g and 0.000928 cm^3^/g, and the average pore diameters are 43.084 Å and 30.8479 nm, respectively (Table S1). In addition, the amount of N_2_ adsorbed by SMA is significantly greater than that adsorbed by PEI-SMA, which indicates that the specific surface area and porosity of PEI-SMA were reduced by the introduction of PEI [28]. Despite the obvious decrease in specific surface area and porosity, PEI-SMA still possessed good adsorption performance, which was mainly attributed to the abundant amine functional groups introduced by grafted PEI. Meanwhile, the successful preparation of PEI-SMA was also indirectly confirmed.

**Figure 2:**
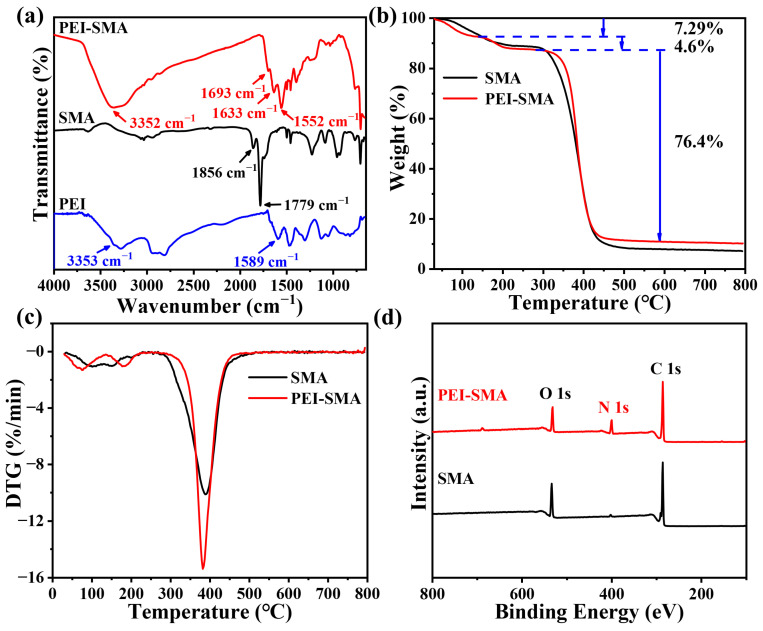
Characterization of SMA and PEI-SMA: (**a**) FT-IR spectra, (**b**) TG curves, (**c**) DTG curves, (**d**) XPS spectra.

The FT-IR spectra of the major functional groups on PEI-SMA, raw SMA, and PEI are analyzed, as shown in Figure 2a. In the FT-IR spectrum curve of SMA, the peaks at 1856 cm^−1^ and 1779 cm^−1^ are ascribed to the characteristic C=O stretching vibration of the anhydride carbonyl group on the SMA [33]. After the PEI grafting modification of SMA, the characteristic anhydride C=O stretching vibration of SMA almost completely disappears, and three new peaks are observed on the FT-IR spectrum curve of PEI-SMA. Among these, the peak at 1693 cm^−1^ is ascribed to C=O stretching vibration of the carboxyl group, while 1633 cm^−1^ (amide I) and 1552 cm^−1^ (amide II) are ascribed to the stretching vibration of C=O and N–C=O in amide groups, respectively, which may result from an aminolysis reaction [34]. In addition, compared to the N–H stretching vibration at 3353 cm^−1^ on the PEI, a broad peak at around 3352 cm^−1^ is also observed for PEI-SMA, which belongs to the stretching vibration of N–H, –OH, and intermolecular hydrogen bonding [35]. These results, therefore, proved that PEI was successfully grafted to SMA.

The thermal stability of PEI-SMA and SMA was investigated by TGA under N_2_ flow and exhibited in Figure 2b. The TG curve of PEI-SMA indicates three stages of weight loss. The first two stages are from 30.1 to 282.6 °C, with about 11.89% weight loss, which could be mainly ascribed to the evaporation of physically adsorbed DMSO and H_2_O. The third stage is from 320.1 to 800.0 °C, with about 76.40% weight loss, which results from the decomposition of the PEI-SMA polymer [36]. This three-stage behavior is also observed in the DTG curves in Figure 2c. Correspondingly, the TGA curve of SMA shows two stages of weight loss. The first stage is from 31.3 to 243.8 °C, with 10.72% weight loss, and the second stage is from 301.3 to 800.0 °C, with 81.16% weight loss. Furthermore, during the polymer decomposition stage, the initial decomposition temperature of SMA is lower than that of PEI-SMA. This indicates that PEI-SMA has a better thermal stability than SMA.

As exhibited in Figure 2d, the XPS spectrum of SMA shows that it mainly contains the elements C (80.17%) and O (18.21%). Additionally, it has a smaller amount of the element N (1.59%) present, which may have resulted from sample contamination by nitrogen in the air. In comparison, after the grafting modification of SMA by PEI, the proportion of the element N significantly increased to about 12.61%. Meanwhile, C and O decreased to 72.88% and 14.26%, respectively, which further proves that PEI has been successfully grafted on the SMA.

### 2.2. Optimization of Synthesis Parameters

**Figure 3:**
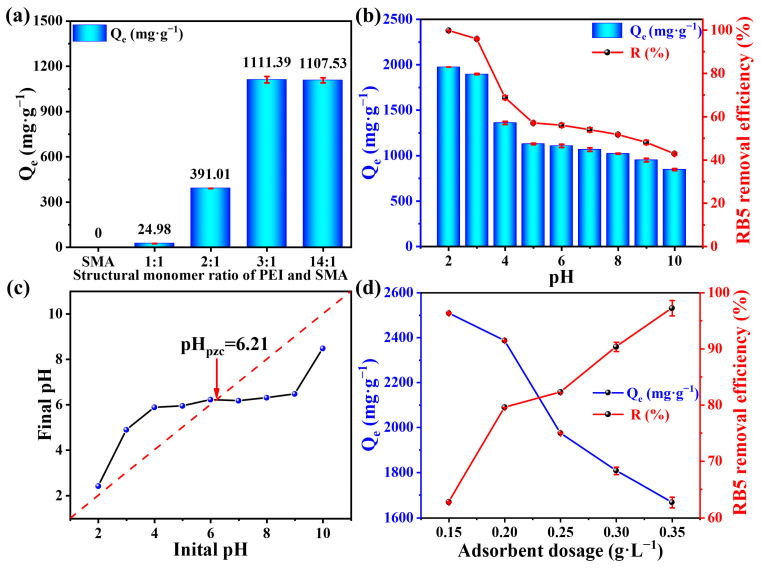
(**a**) The influence of different monomer ratios of PEI and SMA on adsorption capacity of RB5 by PEI-SMA. (RB5 concentration = 300 mg L^−1^, contact time = 1440 min, temperature = 308 K, and original pH); (**b**) the influence of different initial pH values on adsorption capacity and removal efficiency of RB5 by PEI-SMA (contact time = 1440 min, temperature = 308 K, dosage = 0.25 g L^−1^, RB5 concentration = 500 mg L^−1^); (**c**) point of zero charge for PEI-SMA; (**d**) the influence of adsorbent dosage on adsorption capacity and removal efficiency of RB5 by PEI-SMA (contact time = 1440 min, temperature = 308 K, RB5 concentration = 600 mg L^−1^, and pH = 2.0).

In order to introduce more reactive amine groups and obtain the best dye adsorption effect, we explore the influence of different structural monomer ratios of PEI and SMA on the adsorption capacity of PEI-SMA. As shown in Figure 3a, owing to the styrene hydrophobic structures in SMA, RB5 dye was not adsorbed by SMA itself. In addition, for PEI-SMA, as the monomer ratio between PEI and SMA increases from 1:1 to 3:1, the adsorption capacity of RB5 also increases. However, further increasing the monomer ratio to 14:1 does not change the adsorption capacity much. It is noted that the adsorption capacity of RB5 can achieve 1111.39 mg g^−1^ at a monomer ratio of 3:1 between PEI and SMA, which is much higher than those of PEI-SMA at 1:1 (24.98 mg g^−1^) and 2:1 (391.01 mg g^−1^). These facts may result from the increase in the PEI feed ratio, which improves the hydrophilicity of the adsorbent, changing it from being suspended on the liquid surface to being naturally dispersed in the solution [37]. Therefore, we chose the PEI: SMA monomer ratio = 3:1 as the optimal reaction ratio for the synthesis of the PEI-SMA adsorbent.

### 2.3. The Effect of Initial pH

In a certain adsorption system, the dye solution pH plays a critical role by influencing the surface charge of the adsorbents and the ionization of the adsorbate [38]. In Figure 3b, the adsorption behavior of RB5 dye on PEI-SMA shows a strong pH dependence in the solution. The adsorption capacities and percentage removals of RB5 dye on PEI-SMA decreases with the solution pH from pH 2.0 (1974.63 mg g^−1^, 99.86%) to pH 10.0 (847.05 mg g^−1^, 42.84%). These results could be explained by the fact that at lower pH values, the higher H^+^ ions concentration results in the amine groups (−NH_2_) on the PEI-SMA surface possessing a higher protonation degree. This results in increased electrostatic interaction between the positively charged PEI-SMA and the negatively charged RB5 dye, further improving the RB5 dye adsorption capacity. However, at higher pH values, the presence of OH^−^ ions results in the deprotonation of amine groups, decreasing the potential charge between the PEI-SMA adsorbent and RB5 dye molecules, further reducing the adsorption ability [28]. In addition, the non-linear relationship between the initial pH values and the final values are also observed in Figure 3c. When the pH value is below/above 6.21, the initial pH values are lower/higher than the pH value after adsorption, which results from the protonation/deprotonation of amine groups [39]. At pH < 3.75, RB5 (pK_a_ = 3.75) exists in its protonated and ionized form, and PEI-SMA possesses a higher RB5 adsorption capacity due to the strong electrostatic attraction between the fully ionized -SO_3_^−^ group of RB5 and the protonated -NH_3_^+^ group of PEI-SMA. At pH > 3.75, RB5 exists in its basic form, and PEI-SMA can maintain moderate adsorption through anion-exchange interactions with the amino group of PEI-SMA. It is worth mentioning that the PEI-SMA still maintains an RB5 adsorption capacity of 847.05 mg g^−1^ even at pH 10.0, a value that is much higher compared to those previously reported [38]. This may be ascribed to the hydrogen bonding and π–π interactions between the RB5 dye and PEI-SMA. The above results reveal that the RB5 adsorption capacity of PEI-SMA could be maintained higher level over a wide range pH, which is of great interest for practical application.

### 2.4. The Effect of Adsorbent Dosage

The appropriate adsorbent dosage can also significantly influence the adsorption efficiency through affording the available surface area and binding sites [40]. The influence of PEI-SMA adsorbent dosage (0.15–0.35 g L^–1^) on the adsorption capacities and percentage removals of the RB5 dye from the solution is shown in Figure 3d. With the increase in the adsorbent dosage from 0.15 to 0.35 g L^–1^, the percentage removal of the RB5 dye increased from 62.72% to 97.25%. This is mainly ascribed to the increased adsorbent mass, which could provide more active adsorption sites and a larger surface area on PEI-SMA for interacting with the RB5 dye. However, the opposite trend is observed for adsorption capacities, showing a decrease from 2508.97 mg g^−1^ at 0.15 g L^–1^ to 1667.2 mg g^−1^ at 0.35 g L^–1^. This could be interpreted as the limited RB5 dye molecules not being sufficient to fully occupy the adsorption sites, leading to vacant active adsorption sites [41]. It is noted that the removal percentage of RB5 can reach above 97.25% under the 0.35 g L^–1^ adsorbent, indicating that the PEI-SMA could afford adequate active adsorption sites to nearly completely remove RB5 from the solution.

### 2.5. The Effects of Contact Time and Adsorption Kinetics

**Figure 4:**
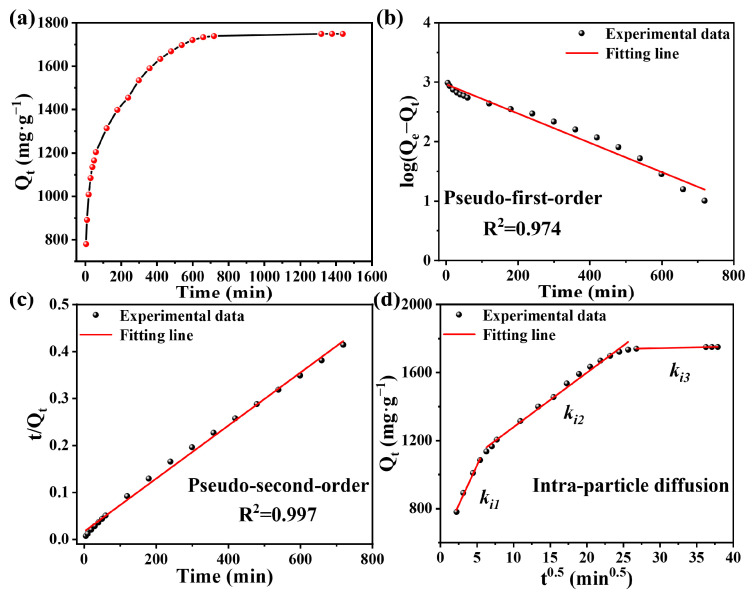
(**a**) The relationship of adsorption capacity with contact time in the adsorption of RB5 by PEI-SMA. Fitting of (**b**) pseudo-first-order kinetic model, (**c**) pseudo-second-order kinetic model, and (**d**) intra-particle diffusion model for adsorption of RB5 by PEI-SMA (concentration = 600 mg L^−1^, dosage = 0.35 g L^−1^, temperature = 308 K, and pH = 2.0).

The effects of contact time on RB5 dye adsorption onto PEI-SMA is exhibited in Figure 4a. In the first 30 min of adsorption, PEI-SMA exhibits the fastest adsorption rate of RB5, which can reach 61.93% of the *q_e_* equilibrium adsorption. This is attributed to the fact that there are adequate adsorption sites on the PEI-SMA surface, and thus more RB5 dye molecules are absorbed at the earlier stage [35]. Subsequently, the rate of adsorption gradually decreases with the consumption of a large number of active adsorption sites. Finally, the equilibrium state is reached, namely, showing equal adsorption/desorption rates [41]. The adsorption equilibrium time and equilibrium adsorption capacity of RB5 for PEI-SMA is 720 min and 1749.19 mg g^−1^, respectively.

The pseudo-first-order (PFO) and pseudo-second-order (PSO) kinetic models and the intra-particle diffusion (IPD) model are applied to assess the experimental data and explore the adsorption mechanism. The model equations are represented by Equations (1)–(3).

PFO kinetic model:

(1) $$\log\left(q_{e}-q_{t}\right)=logq_{e}-\frac{k_{1}t}{2.303}$$

PSO kinetic model:

(2) $$\frac{t}{q_{t}}=\frac{t}{q_{e}}+\frac{1}{k_{2}{q_{e}}^{2}}$$

IPD model:

(3) $$q_{t}=k_{i}t^{1/2}+C$$

where *k*_1_, *k*_2_ (min^−1^) represent the PFO and PSO kinetic model rate constants, respectively, and *q_t_* represents the adsorption capacity at time *t* (min), *C* (mg g^−1^) represents the intercept in IPD plot, and *k_i_* (mg g^−1^ min^−0.5^) represents the IPD rate constant.

The *R*^2^ of the PFO kinetic model (0.974) is smaller than that of the PSO kinetic model (0.997) (Table 1). The data indicate that the PSO kinetic model more accurately describes the adsorption of RB5 dye by PEI-SMA, suggesting the adsorption of RB5 on PEI-SMA mainly involves a chemisorption process. Additionally, the experimental result (1749.19 mg g^−1^) is close to the equilibrium adsorption *q*_e_ computed by the PSO kinetic model (1772.42 mg g^−1^) [36]. Thus, according to the above results, the electrostatic interaction, hydrogen bonding, π–π interactions between PEI-SMA and the RB5 dye play a critical role in the adsorption progress [41].

To explore the effect of RB5 dye molecule diffusion within the pores of PEI-SMA on the whole adsorption process, the IPD model is applied to fit the experimental results, and the relevant fitted results are presented in Table 1. Hence, Equation (5) is applied to determine the rate-limiting step in the adsorption process. Figure 4d reveals that the adsorption kinetic data of PEI-SMA for the RB5 solution could be fitted into three linear regions, and the adsorption rate constants *k_i_*_3_ < *k_i_*_2_ < *k_i_*_1_. The first linear stage of PEI-SMA shows the highest *k_i_*_1_, which is attributed to the adsorption of RB5 by the external boundary layer diffusion on the PEI-SMA surface. The second linear region presents a medium *k_i_*_2_, which corresponds to the interior region diffusion of RB5 in the PEI-SMA. The third stage represents the occurrence of intra-particle diffusion, where RB5 penetrates into the pores. However, such a step proceeds with a very low adsorption rate; the *k_i_*_3_ is almost horizontal due to the small pore volume, as evidenced in the BET analysis [35].

### 2.6. Adsorption Isotherm Evaluation

**Figure 5:**
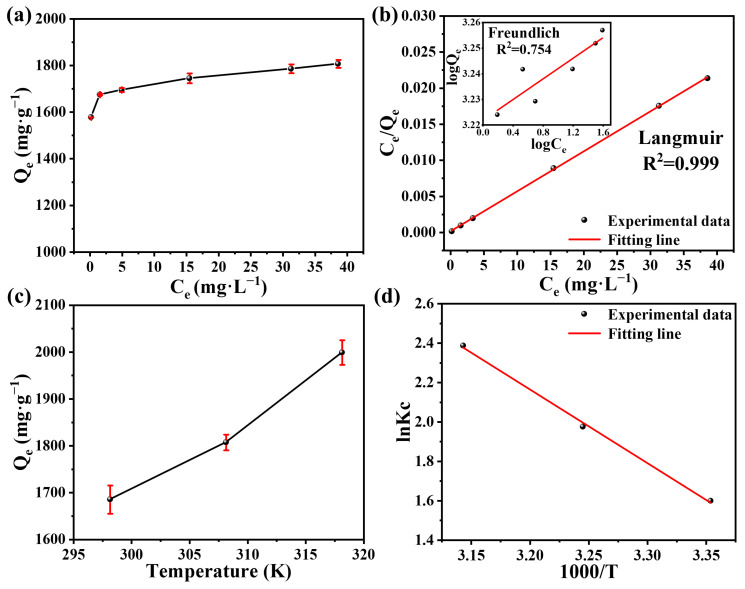
(**a**) The relationship between the *q_e_* of PEI-SMA for RB5 dye and the equilibrium RB5 concentration (*C*_e_) (dosage = 0.35 g L^−1^, contact time = 1440 min, temperature = 308 K, and pH = 2.0); (**b**) the Langmuir and Freundlich adsorption isotherm models for RB5 adsorption by PEI-SMA; (**c**) the relationship between *q*_e_ of PEI-SMA for RB5 dye and the adsorption temperature (concentration = 600 mg L^−1^, dosage = 0.35 g L^−1^, contact time = 1440 min, and pH = 2.0); (**d**) Van ’t-Hoff equation model for RB5 adsorption by PEI-SMA.

The adsorption isotherm can not only be applied to elucidate how RB5 molecules are captured from the aqueous solution by the PEI-SMA adsorbent but also be used to investigate the adsorption mechanisms at specific adsorbent mass, pH, and temperature. Thus, the adsorption isotherm is conducted at different RB5 concentrations (*C*_e_) [28]. As shown in Figure 5a, it is apparent that with the increase in the RB5 concentration (*C*_e_), the equilibrium adsorption capacity (*q*_e_) increased. This result may be ascribed to the fact that during the adsorption process, RB5 molecules gradually occupied the adsorption sites of PEI-SMA, resulting in a decrease in the adsorption potential. On the other hand, as the driving force for the PEI-SMA adsorption of RB5 increased with the initial concentration, more RB5 dye molecules could combine with the active adsorption sites on the PEI-SMA surface, resulting in higher adsorption capacities. Meanwhile, at higher initial dye concentrations, more adsorbates are expected to compete for a fixed number of active adsorption sites, leading to the saturation of adsorption sites and an increase in adsorption capacity [40].

Langmuir and Freundlich isotherm models are applied to fit the adsorption data obtained. The equations are listed as Equations (4)–(6):

Langmuir isotherm model is the following:

(4) $$\frac{C_{e}}{q_{e}}=\frac{C_{e}}{q_{m}}+\frac{1}{q_{m}K_{L}}$$

(5) $$R_{L}=\frac{1}{1+K_{L}C_{0}}$$

where *C*_0_, *C*_e_ (mg L^−1^) represent the initial and equilibrium concentrations in the dye solution, respectively; *q*_e_ and *q*_m_ (mg g^−1^) are the adsorption capacity at equilibrium and maximum, and *K*_L_ represents the Langmuir adsorption constant. The *R*_L_ value represents either favorable or unfavorable adsorption isotherms: unfavorable when *R*_L_ > 1, favorable when 0 < *R*_L_ < 1.

Freundlich isotherm model:

(6) $$logq_{e}=logK_{F}+\frac{1}{n}logC_{e}$$

*K*_F_ represents the Freundlich adsorption constant, and *n* represents the adsorption intensity.

Comparing the *R*^2^ values of the Langmuir model (0.999) and the Freundlich model (0.754) in Table 2, it is evident that the adsorption process of PEI-SMA on RB5 is suited to be described by the Langmuir isotherm model, suggesting that the adsorption process involves single-molecular layer adsorption on the PEI-SMA surface. The *R*_L_ = 0.00044 (0 < *R*_L_ <1) indicates that the adsorption process of RB5 onto PEI-SMA is favorable [39]. The experimental data (1749.19 mg g^−1^) of PEI-SMA-adsorbed RB5 are similar to the maximum adsorption capacity computed by the Langmuir isotherm model (1809.30 mg g^−1^). As shown in Table 3, the PEI-SMA in this work shows superior performance to other PEI-based adsorbents in terms of maximum adsorption capacity [41,42,43,44,45,46].

### 2.7. Adsorption Thermodynamics

Temperature is also an important factor during the adsorption process, which can influence the diffusion rate of the dye molecules at the external interface and within the pores of the adsorbent [28]. In Figure 5c, as the temperature increases from 298 K to 318 K, the adsorption capacity of PEI-SMA for RB5 presents an obvious upward trend: the adsorption capacity increases from 1685.37 mg g^−1^ to 1998.97 mg g^−1^. This suggests that the adsorption of PEI-SMA for RB5 is an endothermic process. This can be mainly ascribed to the fact that the average kinetic energy of RB5 increases with increasing temperature, which results in increased collision and binding with the active adsorption sites [35]. The adsorption thermodynamics parameters of RB5 adsorbed by PEI-SMA are assessed by the Van ’t-Hoff equation model, and the fitting line is exhibited in Figure 5d. The equations used are listed as Equations (7)–(9):

(7) $$K_{c}=\frac{q_{e}}{C_{e}}$$

(8) $$∆G^{\theta }=-RTlnK_{c}$$

(9) $$lnK_{c}=\frac{{∆S}^{\theta }}{R}-\frac{{∆H}^{\theta }}{RT}$$

*K*_c_ represents the equilibrium constant; *R* represents the gas constant; Δ*G^θ^*, Δ*H^θ^*, and Δ*S^θ^* are the Gibbs free energy, enthalpy, and entropy, respectively.

The calculated thermodynamic parameters are shown in Table 4. The calculated Δ*G^θ^* values are negative, and their absolute values increase with increasing temperature. This indicates that increasing the temperature could increase the adsorption spontaneity of the RB5 dye by PEI-SMA. The positive Δ*H^θ^* value denotes the endothermic nature of the adsorption process for RB5 dye. The positive Δ*S^θ^* value indicates interfacial disorder between the solid adsorbent and the aqueous solution. This further supports the possibility that the process occurs due to an increase in entropy.

### 2.8. Selective Adsorption Behavior

**Figure 6:**
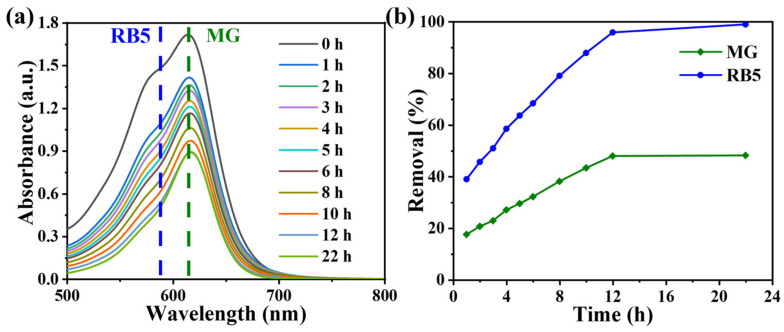
UV-vis spectra of (**a**) RB5/MG mixed solution with different contact time (RB5 and MG concentration = 600 mg L^−1^, respectively; V = 200 mL, dosage = 35 mg, contact time = 1440 min, temperature = 308 K, and pH = 2.0). (**b**) The relationship between the removal percentage of RB5 and MG in the mixed dye solutions and time.

The time-absorbance curves of binary mixed dye solutions of RB5 and Malachite Green (MG) dyes by UV-vis spectra are shown in Figure 6a. Throughout the adsorption process, the absorbance at the maximum absorption wavelength of RB5 (596 nm) decreased faster than that of MG (617 nm). This phenomenon reveals that the PEI-SMA priorly adsorbs the anionic RB5 dye. To further prove that the PEI-SMA can selectively adsorb anionic RB5 dye rather than cationic MG dye, the corresponding percentage removal rate-time curves are also carried out. In Figure 6b, the adsorption of the anion-cation binary mixed system reaches equilibrium after approximately 12 h of adsorption, and the removal rates of MG and RB5 can achieve 48.22% and 99.07% at 22 h, respectively. During the adsorption process, compared to MG, the adsorbent exhibits higher adsorption and removal rates for RB5. That is to say, the adsorbent shows better adsorption selectivity for the anionic dye RB5 compared to the cationic dye MG. This is mainly because the amino groups on PEI-SMA polymer can interact with the anionic dye RB5 through electrostatic interactions under pH = 2; however, it rejects the cationic dye MG [47,48].

Figure S2a shows the time-absorbance curves of the binary mixed dye solutions of RB5 and MO under UV-vis spectra. Throughout the adsorption process, the absorbance at the maximum absorption wavelength of MO (498 nm) decreased faster than that at the maximum absorption wavelength of RB5 (597 nm). This phenomenon indicates that PEI-SMA can preferentially adsorb anionic MO dye. In order to further investigate the adsorption of RB5 and MO dyes by PEI-SMA, the corresponding percentage removal rate-time curves were plotted. In Figure S2b, the removal rate of MO and RB5 by PEI-SMA in the anionic binary system reached 62.00% and 27.71%, respectively, after 1 h of adsorption. This is mainly due to the smaller molecular weight and volume of MO compared to RB5, which leads to faster diffusion and binding with adsorption sites in the early stages of adsorption [40,41]. After 26 h of adsorption, the removal rates of MO and RB5 reached 91.21% and 89.16%, respectively, indicating that PEI-SMA had a good adsorption effect on both anionic dyes.

### 2.9. Effects of Coexisting Ions

**Figure 7:**
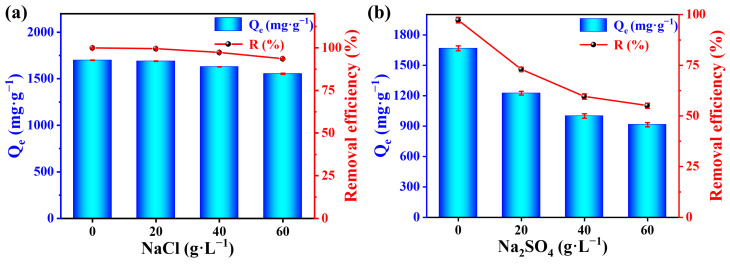
Effect of coexisting ions (**a**) NaCl and (**b**) Na_2_SO_4_ on adsorption of RB5 by PEI-SMA (concentration = 600 mg L^−1^, dosage = 0.35 g L^−1^, contact time = 1440 min, temperature = 308 K, and pH = 2.0).

In the printing and dyeing industry, salts such as Na_2_SO_4_ and NaCl are often used as dyeing promoters for cotton fibers dyed with reactive dyestuffs and as retarding agents for silk and wool dyed with acid or cationic dyes [49]. As a result, dyeing effluents contain large amounts of SO_4_^2–^ and Cl^–^ ions. For this reason, the adsorption capacity and removal rate of RB5 dye in the presence of SO_4_^2–^ and Cl^–^ ions with different mass concentrations was explored to assess the adsorption ability of the PEI-SMA adsorbent. In Figure 7a,b, the adsorption capacity and removal rate of RB5 dye by PEI-SMA adsorbent decrease with the increase in SO_4_^2–^ and Cl^–^ ion concentrations. This is because SO_4_^2–^ and Cl^–^ ions compete with the SO_3_^–^ group of the RB5 molecule, competing with the –NH_3_^+^ site on the surface of the PEI-SMA adsorbent. The experimental results show that when the concentration of Cl^–^ and SO_4_^2–^ ions is 60 g L^–1^, the adsorption capacity decreases to 1552.63 and 913.62 mg g^−1^, respectively. It was also observed that at the same mass concentrations of SO_4_^2–^ and Cl^–^ ions, SO_4_^2–^ ions interfered more severely in the adsorption of RB5 compared to Cl^–^ ions. This may be due to the fact that the SO_4_^2–^ ion is a divalent negative ion with stronger electrostatic interactions with the protonated amine group, resulting in a more significant decrease in adsorption capacity and removal rate of RB5 dye [30].

### 2.10. Regeneration of Adsorbent and Continuous Sorption Assessment

**Figure 8:**
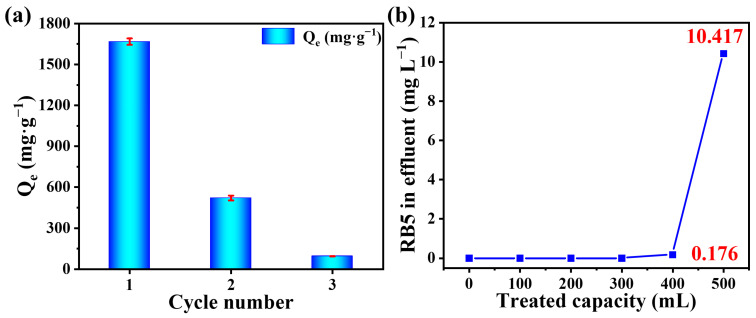
(**a**) Regeneration performance and reusability of PEI-SMA for RB5 adsorption (concentration = 600 mg L^−1^, dosage = 0.35 g L^−1^, contact time = 1440 min, temperature = 308 K, and pH = 2.0). (**b**) Continuous flow history for RB5 dye removal (concentration = 100 mg L^−1^, dosage = 0.25 g L^−1^, and pH = 2.0).

To study the regeneration performance of PEI-SMA, the regeneration cycle experiments of PEI-SMA for RB5 are investigated using 1 M NaOH and anhydrous ethanol as eluents, and the results are exhibited in Figure 8a. The adsorption capacity of PEI-SMA for RB5 dramatically decreased from 1667.2 mg g^−1^ to 520.83 mg g^−1^ after the first regeneration, and the adsorption capacity further decreased to 94.7 mg g^−1^ after the second regeneration. This indicates that the PEI-SMA adsorbent has good adsorption stability for the RB5 dye, while its cyclic regeneration adsorption performance for the RB5 dye is poor. This may be due to the fact that the pores formed by the winding and stacking of PEI-SMA polymer chains are relatively small, while the volume of the RB5 molecule is relatively large. As a result, the adsorbed RB5 molecules find it difficult to escape from the adsorbent [50]. Moreover, there are multiple sulfonic acid ions on RB5 molecule, which may bind to multiple amine sites on the PEI-SMA polymer chain during the desorption process. In addition, RB5 is a reactive dye, which could react with the amine or carboxyl groups on the surface of PEI-SMA to form covalent bonds, thus becoming immobilized on the adsorbent surface [28]. The high adsorption capacity and stability of PEI-SMA for RB5 may make it a candidate for use as a dyeing assistant or color masterbatch dispersant.

A continuous adsorption experiment was performed to assess the applicability of PEI-SMA in treating simulated wastewater containing 100 mg L^−1^ of RB5 dye [51]. As shown in Figure 8b, when the treated capacity of RB5 dye is less than 300 mL, the RB5 dye can not be detected in the wastewater by a UV-vis spectrophotometer. When the treated capacity was 400 mL, the residual amount of RB5 dye in the effluent was only 0.176 mg L^−1^. The adsorption capacities reached 1178.52 mg g^−1^ and 1554.46 mg g^−1^, and the removal rates reached approximately 100% and 99.82%, respectively. In other words, 1 g of PEI-SMA adsorbent was able to treat 16 L of RB5 dye solution (100 mg L^−1^), resulting in residual RB5 dye levels of less than 0.176 mg L^−1^.

### 2.11. Adsorption Mechanism

**Figure 9:**
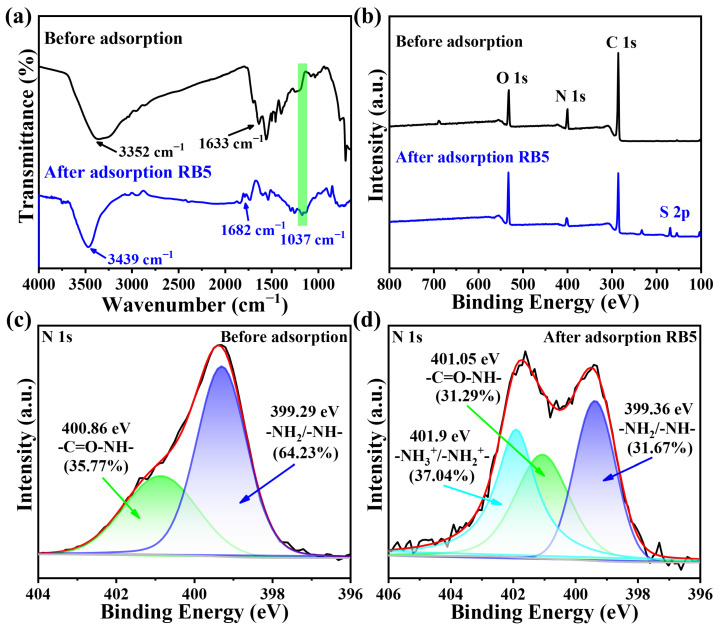
(**a**) FT-IR spectra and (**b**) XPS survey spectra of PEI-SMA, RB5 adsorbed PEI-SMA, respectively; (**c**,**d**) the peak splitting fitting diagram of N 1 s before and after adsorption of RB5, respectively.

To understand the RB5 dye adsorption mechanism on PEI-SMA, the FT-IR and XPS were used to characterize the PEI-SMA before and after the adsorption of RB5 dyes. As shown in Figure 9a, compared with the FT-IR peaks intensity of initial PEI-SMA, the peak intensity for PEI-SMA decreases after the adsorption process, suggesting that the RB5 molecules are adsorbed by PEI-SMA [41]. Furthermore, after adsorbing RB5, a new peak is observed at about 1037 cm^–1^, which can be ascribed to the O=S=O stretching vibration in RB5, indicating that PEI-SMA successfully adsorbed the RB5 dyes [52]. After the adsorption RB5, the stretching and bending vibration absorption peaks of –NH_2_/–NH groups on pristine PEI-SMA at 3352 cm^–1^ and 1633 cm^–1^ show a red shift, suggesting that the –NH_2_/–NH groups took part in the adsorption reaction [41].

The XPS spectra of PEI-SMA before and after adsorption of RB5 are shown in Figure 9b. S is the characteristic element of RB5 molecules. Compared with the XPS spectrum of pristine PEI-SMA, after adsorption of RB5, a strong peak at 168.08 eV belonging to S2p emerges in the XPS spectra of PEI-SMA, indicating that RB5 molecules are successfully adsorbed on PEI-SMA [28]. Figure 9c,d show the peak splitting fitting of N 1 s before and after PEI-SMA adsorbs the RB5 molecules, respectively. As shown in Figure 9c, 400.86 eV and 399.29 eV in PEI-SMA are ascribed to –C=O–NH– and –NH_2_/–NH– groups, respectively [38]. Compared with Figure 9c, after the adsorption of RB5 molecules, the peaks corresponding to –C=O–NH– and –NH_2_/–NH– groups in PEI-SMA shift toward higher binding energy, moving to 401.05 eV and 399.36 eV, respectively. This reveals the formation of hydrogen bonding [39]. After adsorption of RB5, the proportion of –NH_2_/–NH– groups on PEI-SMA significantly decreased, and new –NH_3_^+^/–NH_2_^+^– groups appeared, indicating an electrostatic interaction between PEI-SMA and RB5 molecules [35].

**Figure 10:**
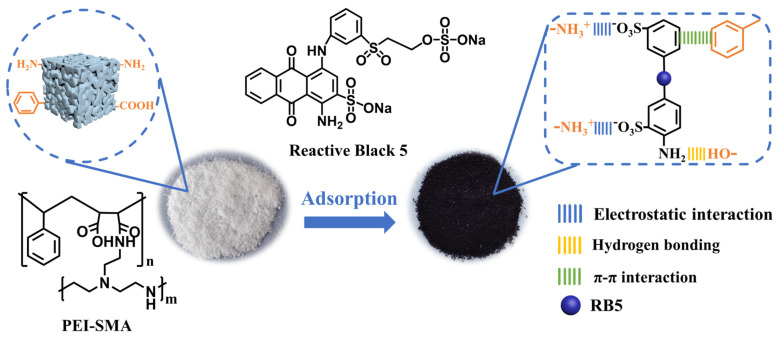
Possible mechanism and photos of RB5 adsorption by PEI-SMA.

Based on the above analysis, the dyes adsorption mechanisms for the PEI-SMA could be concluded, as shown in Figure 10. The electrostatic interaction, hydrogen bonding and π–π interaction mainly participate into the adsorption of RB5 by the PEI-SMA.

## 3. Materials and Methods

### 3.1. Materials

Styrene-maleic anhydride copolymer (SMA, Mw 28000), Malachite Green (MG), dimethyl sulfoxide (DMSO), and Reactive Black 5 (RB5) were obtained by Macklin Biochemical Co., Ltd. (Shanghai, China). Polyethyleneimine (PEI, Mw 600) was supplied by Aladdin Chemistry Co., Ltd. (Shanghai, China). HCl and NaOH were obtained from Sinopharm Chemical Reagent Co., Ltd. (Shanghai, China). All reagents are analytical reagent pure and have not been further purified. Cellulose dialysis membranes (molecular weight cut-off 14 kDa) were obtained from Viskase (Chicago, IL, USA).

### 3.2. Preparation of PEI-SMA

**Figure 11:**
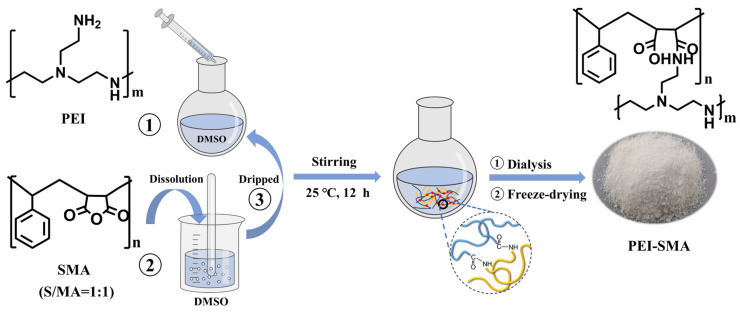
Schematic diagram of preparing the PEI-SMA adsorbent.

Figure 11 shows the preparation process of PEI graft-modified SMA polymer (PEI-SMA) adsorbent and the chemical structure of SMA and PEI. Firstly, 0.5 g of PEI and 0.8 g of SMA were dissolved in 20 mL of DMSO, respectively. Then, the DMSO solution with dissolved SMA was added dropwise to the DMSO solution with dissolved PEI, and magnetic stirring was turned on at the same time to make the reaction proceed sufficiently. The reaction was carried out at 25 °C with stirring for 12 h. Then, the reaction compound was dialyzed with cellulose dialysis membranes in deionized water for 24 h to wash out the residual DMSO and unreacted PEI [34]. Finally, the samples were dried by vacuum freeze-drying to afford PEI-SMA.

### 3.3. Characterization

Fourier transform infrared (FT-IR) spectral analysis of the samples was carried out on a Nicolet iS10 FT-IR spectrometer (Nicolet, Madison, WI, USA) using the standard KBr disk method. The microscopic morphology and elemental compositions of samples were obtained using scanning electron microscopy (SEM, FEI-Nova Nano 230, Hillsboro, OR, USA) with an energy-dispersive X-ray spectrometer (EDS). The ASAP2460 (Micromeritics, Norcross, GA, USA) automatic specific surface area and pore size analyzer was utilized to determine the specific surface area and porosity parameters of the samples using the Brunauer-Emmett-Teller (BET) method and the Barrett-Joyner-Halenda (BJH) method, respectively. The weight loss curves of the samples were obtained by thermogravimetric analysis (TGA, Netzsch-STA449F5 Jupiter, Selb, Germany) from 30 to 800 °C under a nitrogen atmosphere. The absorbance of the dye solution was analyzed by a UV-vis spectrophotometer (UV-2600, Shimadzu, Kyoto, Japan). X-ray photoelectron spectroscopy (XPS, Thermo Fisher Nexsa, Hillsboro, OR, USA) was performed to assess the change in element composition of the samples before and after adsorption.

### 3.4. Adsorption Experiments

The adsorption performance of PEI-SMA adsorbent was assessed by the batch adsorption method. The pH of the dye solution was regulated with 1 M HCl and 1 M NaOH. The effects of solution pH values of RB5 (2.0–10.0), adsorbent dosage (0.15–0.35 g L^–1^), initial concentrations of RB5 (590–670 mg L^–1^), contact time (0–1440 min) with RB5, and temperature of the adsorption system (298–318 K) on the adsorption performance were studied, respectively. The reusability of the PEI-SMA was studied by using 1 M NaOH and anhydrous ethanol as the desorbents. The dye equilibrium adsorption capacity (*q*_e_, mg g^−1^) and the dye removal percentage were computed according to Equations (10) and (11), respectively.

(10) $$q_{e}=\frac{\left(C_{0}-C_{e}\right)}{m}\times V$$

(11) $$R\left(\%\right)=\frac{\left(C_{0}-C_{e}\right)}{C_{0}}\times 100$$

where *C*_0_, *C*_e_ (mg L^−1^) represent the initial and equilibrium concentration in the dye solution, respectively, *V* (L) represents the volume of the dye solution, and *m* (g) represents the adsorbent mass.

## 4. Conclusions

In conclusion, two commercially available polymers, SMA and PEI, were used to prepare the PEI-SMA polymer for dye adsorption through a one-step acylation reaction. The optimal feed ratio of PEI/SMA for PEI-SMA synthesis was determined to be 3:1. The effects of initial pH, adsorbent dosage, contact time, RB5 concentration, and temperature on the adsorption performance of PEI-SMA were systematically studied. The adsorption process of RB5 on PEI-SMA was more consistent with the Langmuir isotherm model and the pseudo-second-order kinetic model, indicating a single-layer adsorption, which was mainly controlled by chemisorption. According to the Langmuir isotherm model, the maximum adsorption capacity of PEI-SMA for RB5 was 1809.30 mg g^−1^. The thermodynamic analysis suggests that the adsorption process was a spontaneous endothermic process. The adsorption of RB5 by PEI-SMA was mainly due to electrostatic interaction, hydrogen bonding, and π–π interaction between RB5 and PEI-SMA. Although coexisting ions, such as Cl^–^ and SO_4_^2–^, significantly reduce the adsorption capacity of PEI-SMA for RB5, its adsorption capacity can still reach as high as 1552.63 mg g^–1^ at a concentration of 60 g L^–1^ of coexisting Cl^–^. The mixed adsorption experiments with cationic MG dyes show that PEI-SMA exhibits better adsorption selectivity for anionic dyes. PEI-SMA has both high adsorption capacity and stability for RB5 dye, making it promising for use as a printing and dyeing assistant or polymer dye.

## Figures and Tables

**Table 1:** Adsorption kinetics model parameters of RB5 dye adsorption by PEI-SMA.

Kinetic Model	Parameter	RB5
PFO	*k*_1_ (min^−1^)	5.69 × 10^−3^
	*q*_e_ (mg g^−1^)	919.62
	*R* ^2^	0.974
PSO	*k*_2_ (min^−1^)	2.06 × 10^−5^
	*q*_e_ (mg g^−1^)	1772.42
	*R* ^2^	0.997
IPD	*k_i_*_1_ (mg g^−1^ min^−0.5^)	93.32
	*R* ^2^	0.986
	*k_i_*_2_ (mg g^−1^ min^−0.5^)	31.40
	*R* ^2^	0.989
	*k_i_*_3_ (mg g^−1^ min^−0.5^)	0.94
	*R* ^2^	0.979
Experimental *q*_e_ (mg g^−1^)		1749.19

**Table 2:** Adsorption isotherm model parameters of RB5 dye adsorption by PEI-SMA.

Adsorbent		Langmuir				Freundlich	
	*K*_L_	*q*_m_	*R*_L_	*R* ^2^	*K*_F_	1*/n*	*R* ^2^
PEI-SMA	3.76	1809.30	0.00044	0.999	1666.79	0.020	0.754

**Table 3:** The adsorption capacity of RB5 by different PEI-based adsorbents.

Adsorbent	*q_max_* (mg g^–1^)	pH	Temperature	Equilibrium Time	Reuse Times	Reference
PEI-STL PEI-CW	71.9 77.52	3 7	318 K 303 K	200 m 180 m	/ /	[41] [40]
PEI-OzHC MCPEI PEI–PVC fiber PEI-CSB PEI-CB PEI-PVCF fiber PEI-SMA	182.7 330.0 314.4 413.23 709.27 1265.0 1809.3	2 7 2 6 6 7 2	318 K 300 K 298 K 303 K 303 K 298 K 308 K	360 m 180 m 360 m 1440 m 1440 m 320 m 720 m	/ 5 / / / 5 3	[42] [43] [44] [45] [45] [46] This study

STL: spent tea leaves, CW: coffee waste, OzHC: ozone oxidized hydrochar, MCPEI: magnetic nanoparticle-cellulose, PVC: polyvinyl chloride, CSB: chitosan hydrogel beads by sodium dodecyl sulphate, CB: chitosan hydrogel beads by alkali, PVCF: polyvinyl chloride fiber.

**Table 4:** Thermodynamic parameters of PEI-SMA for the adsorption of RB5 dye.

Adsorbent	Δ*H* (kJ/mol)	Δ*S* (J/mol K)	Δ*G* (kJ/mol)	Temperature (K)
			−3.96	298
PEI-SMA	31.10	117.51	−5.06	308
			−6.31	318

## Data Availability

All data obtained in this study are included in this paper.

## Supplementary Materials

The following supporting information can be downloaded at: https://www.mdpi.com/article/10.3390/molecules29081887/s1, Figure S1: Characterization of N_2_ adsorption–desorption isotherms: (a) SMA, (b) PEI-SMA; Figure S2: UV-vis spectra of (a) RB5/MO mixed solution with different contact time (RB5 and MO concentration = 600 mg L^−1^, respectively; V = 200 mL, dosage = 35 mg, contact time = 1560 min, temperature = 308 K, and pH = 2.0); (b) The relationship between the removal percentage of RB5 and MO in the mixed dye solutions and time; Table S1: The BET specific surface area, pore volume, average pore diameter, meso- and micropore volume data of SMA and PEI-SMA.
