# Supplementary material for: One-Step Synthesis of Polyethyleneimine-Grafted Styrene-Maleic Anhydride Copolymer Adsorbents for Effective Adsorption of Anionic Dyes
Source: Molecules. 2024 Apr 21;29(8):1887. doi: 10.3390/molecules29081887 (PMC11054579; doi:10.3390/molecules29081887)
Supplement: Supplementary file 1 [file molecules-29-01887-s001.zip › molecules-2954513-supplementary.pdf]

One-step synthesis of polyethyleneimine grafted styrene-maleic anhydride copolymer adsorbents for effective adsorption of anionic dyes

Yao Xu[a], Qinwen Wang[a], Yuanbo Wang[b], Falu Hu*[a], Bin Sun[a, c], Tingting Gao*[a,c], Guowei Zhou[a]

[a] *Key Laboratory of Fine Chemicals in Universities of Shandong, Jinan Engineering Laboratory for Multi-scale Functional Materials, School of Chemistry and Chemical Engineering, Qilu University of Technology (Shandong Academy of Sciences), Jinan 250353, China*

[b] *Shandong Land and Space Ecological Restoration Center, Jinan, China*

[c] *Shandong Laboratory of Advanced Materials and Green Manufacturing at Yantai, Yantai, China*

*Corresponding author at: Daxue Road, Western University Science Park, Jinan 250353, Shandong, China. Tel: +86-13573103906

E-mail address: faluhu@qlu.edu.cn; ttgao@qlu.edu.cn

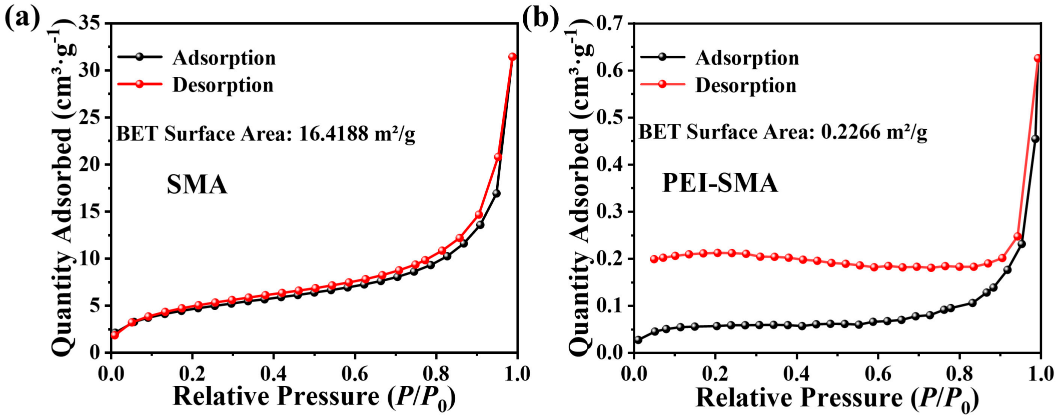

Figure S1. Characterization of N_2 adsorption–desorption isotherms: (a) SMA, (b) PEI-SMA.

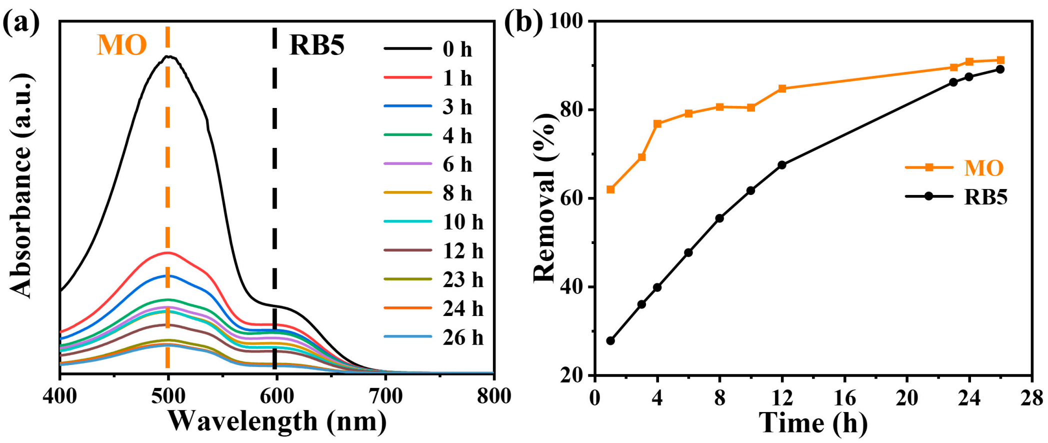

Figure S2. UV-vis spectra of (a) RB5/MO mixed solution with different contact time (RB5 and MO concentration = 600 mg L^{-1}, respectively; V = 200 mL, dosage = 35 mg, contact time = 1560 min, temperature = 308 K, and pH = 2.0); (b) The relationship between the removal percentage of RB5 and MO in the mixed dye solutions and time.

Table S1. The BET specific surface area, pore volume, average pore diameter, meso- and micropore volume data of SMA and PEI-SMA.

Parameters	SMA	PEI-SMA
surface area	16.4188 m^2/g	0.2266 m^2/g
pore volume	0.014067 cm^3/g	0.000928 cm^3/g
average pore diameter	43.084 Å	30.8479 nm
mesopore volume	0.005105 cm^3/g	0.000605 cm^3/g
micropore volume	0.001723 cm^3/g	0.000004 cm^3/g
